# Measurements of thermophysical properties of solid and liquid NIST SRM 316L stainless steel

**DOI:** 10.1007/s10853-019-04261-6

**Published:** 2020

**Authors:** Peter Pichler, Brian J. Simonds, Jeffrey W. Sowards, Gernot Pottlacher

**Affiliations:** 1 Institute of Experimental Physics, Graz University of Technology, NAWI Graz, Petersgasse 16, 8010 Graz, Austria; 2 National Institute of Standards and Technology, 325 Broadway, Boulder, CO 80305, USA; 3 Present address: NASA Marshall Space Flight Center, Huntsville, AL 35812, USA

## Abstract

In this work, we perform high accuracy measurements of thermophysical properties for the National Institute of Standards and Technology standard reference material for 316L stainless steel. As these properties can be sensitive to small changes in elemental composition even within the allowed tolerances for an alloy class, by selecting a publicly available standard reference material for study our results are particularly useful for the validation of multiphysics models of industrial metal processes. An ohmic pulse-heating system was used to directly measure the electrical resistivity, enthalpy, density, and thermal expansion as functions of temperature. This apparatus applies high current pulses to heat wire-shaped samples from room temperature to metal vaporization. The great advantage of this particular pulse-heating apparatus is the very short experimental duration of 50 μs, which is faster than the collapse of the liquid wire due to gravitational forces, as well as that it prevents any chemical reactions of the hot liquid metal with its surroundings. Additionally, a differential scanning calorimeter was used to measure specific heat capacity from room temperature to around 1400 K. All data are accompanied by uncertainties according to the guide to the expression of uncertainty in measurement.

## Introduction

The National Institute of Standards and Technology (NIST) standard reference material (SRM) for 316L stainless steel (1155a) has recently been used for studies of intense laser light coupling in metal to provide data for the validation of multiphysics models of industrial laser processes like welding, cutting, and additive manufacturing [1]. In order to reduce the costs associated with empirical trial-and-error production development, manufacturers are increasingly looking to multiphysics computer simulations to more rapidly optimize process parameters. Generally, these models simulate the laser heating of metal followed by heat flow and fluid transport of the solid/molten metal system in order to predict the evolution of the fusion zone and surrounding heat-affected zone. Therefore, in addition to laser light coupling they also require many thermophysical material properties over a very wide temperature range across solid and liquid phases. Ideally, modelers would be able to find accurate thermophysical property values with known uncertainties spanning the wide temperature range necessary for the exact alloy composition they are modeling. However, due to the limited amount of data available modelers often resort to using values for materials of similar, but not exact, composition to that which they are studying, and extrapolate for values at temperatures not found in the literature.

**Figure 1:**
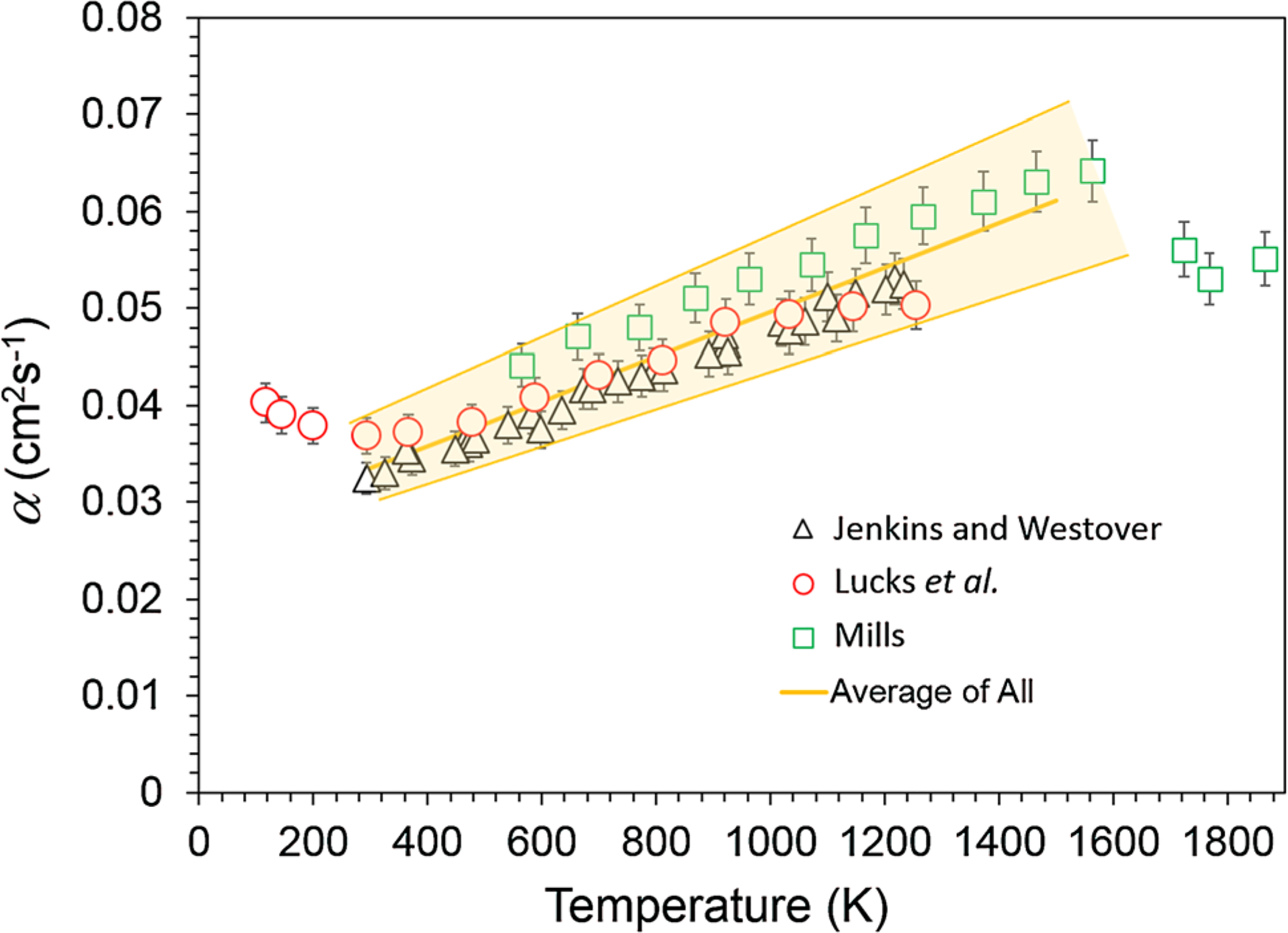
Experimental values for the thermal diffusivity of 304 stainless steel. The solid line represents an average of all values between room temperature and the melting point. The shaded area represents an uncertainty of 15%.

Thermal diffusivity is the relevant material property used in the heat equation to model transient heat conduction during, e.g., welding and additive manufacturing. Figure 1 shows experimental values for thermal diffusivity and their uncertainty, as a function of temperature for the common industrial alloy 304 stainless steel. These data were taken from three different laboratories using different feedstocks for 304 stainless steel [2–4]. Even though these materials meet the composition requirements for 304 stainless steel, the allowed tolerance range for the primary constituent elements Fe, Cr, Ni, and Mo [5] can lead to variations in the microstructure that the thermal diffusivity is sensitive to [6]. The solid curve is an average of all values between room temperature and the melting point. The shaded band spans ±15%, which encompasses all experimental values and their uncertainties. This illustrates the dilemma for the modeler which is to decide which data set to use.

**Figure 2:**
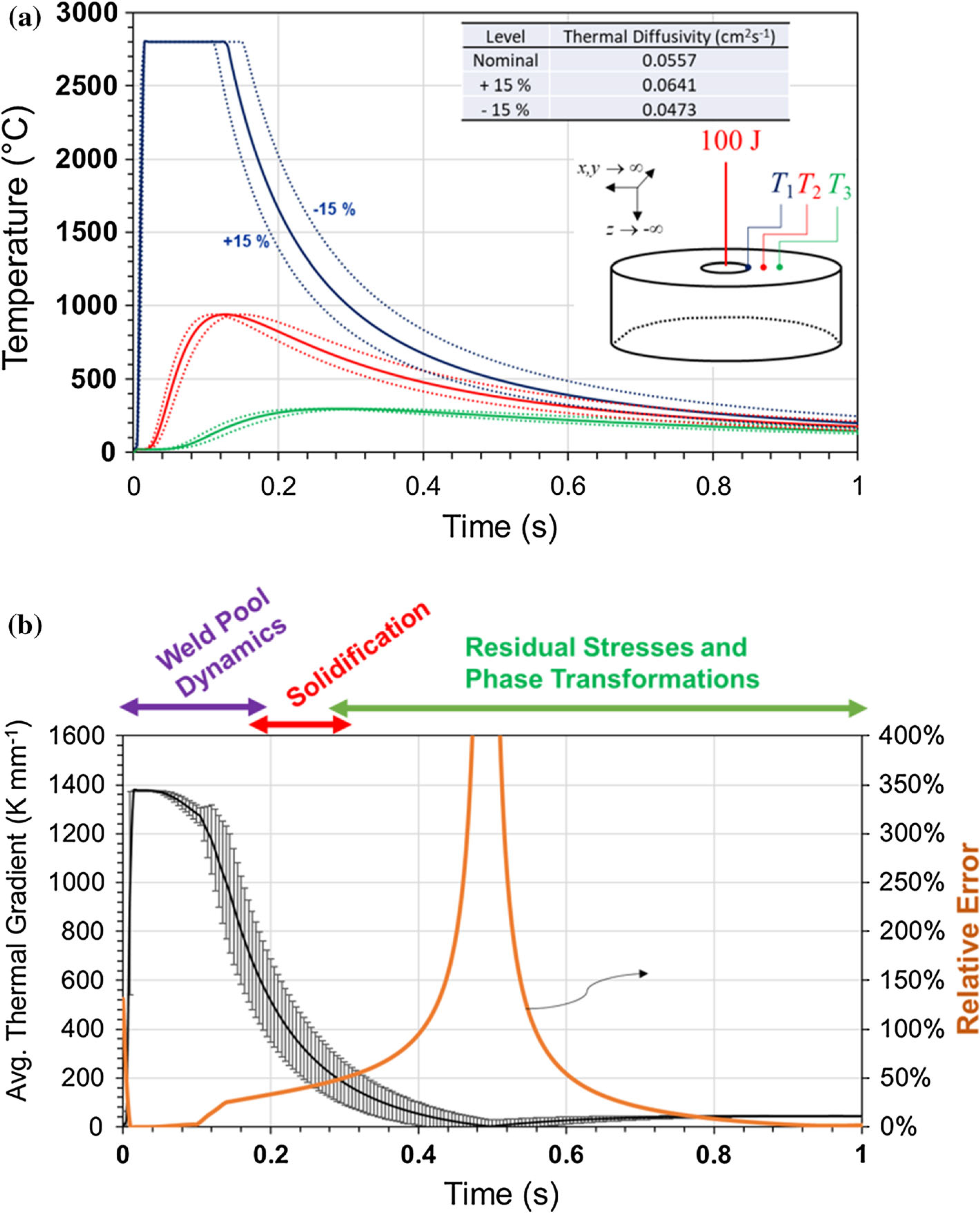
Analytical solutions to the heat transfer equation at three points along the surface of a semi-infinite body after an instantaneous heat load of 100 J are given as solid lines in **a**. An inset shows the location of the three temperature probes, *T*1, *T*2, and *T*3. The dashed lines show the temperature range resulting from a 15% variance in thermal diffusivity. The average thermal gradient at the material surface is computed and plotted as the solid black curve in **b**. The error bars indicate the spread of values from the computed temperature ranges in **a**. The right axis in **b**. shows the relative error caused by the thermal diffusivity-induced uncertainty in temperature.

To illustrate the effect that this can have on a model prediction, we perform a simple numerical exercise. We model the temperature field resulting from an instantaneous heat load of 100 J applied as a point source to one surface of a semi-infinite body at time *t* = 0. This is a very simplified approximation of a laser spot weld, and the temperature field in three spatial dimensions *x*, *y*, and *z* is given by

(1) $$T(x,y,z,t)=\frac{Q}{{\left(4\pi \alpha t\right)}^{3/2}}\exp\left(-\frac{x^{2}+y^{2}+z^{2}}{4\alpha t}\right),$$

where *Q* is the heat load (100 J), *t* is time, and a is the thermal diffusivity. For α, we use a nominal value of 0.0557 cm^2^ s^−1^ which is found by taking an average from Fig. 1. Equation (1) is solved analytically for the time-dependent temperature at points on the surface (*y*, *z* = 0 mm) of the semi-infinite body at three distances (*x* = 1 mm; 2 mm, and 3 mm) away from the location of the heat load (*x*, *y*, *z* = 0) as shown in the inset of Fig. 2a. These results are plotted as solid curves in Fig. 2a. Next, we repeat the calculations but for values of α that represent the spread found in the literature, approximately ±15%. These results are given as dashed curves in Fig. 2a. It is assumed that the temperature of the molten weld pool cannot exceed the boiling point of stainless steel (assumed here to be 2800 K).

From these curves, we derive the average thermal gradient, *G*, according to

(2) $$G=\frac{\Delta T}{\left(x_{T_{3}}-x_{T_{1}}\right)}.$$

The thermal gradient is important for development of metallurgical phenomena such as residual stress and phase transformations. Stainless steel alloy 316, in particular, (which is the SRM used herein) can exhibit a strong tendency toward a shift in solidification mode due to increased thermal gradient, resulting in an increase in weld cracking sensitivity [7]. *G* is plotted in Fig. 2b. on the left ordinate as a solid black line found using the nominal value of α. The error bars represent the deviation that occurs due to the temperature range resulting from different values of a. This range is then used to determine the time-dependent relative error in the average thermal gradient, which is plotted on the right ordinate. Its value exceeds several hundred percent with the peak value precisely in the region where the final weld properties are determined. In these calculations, we use a single, non-temperature-dependent value for α for simplicity. However, using a temperature-dependent value would not change the relative error as whichever temperature-dependent values chosen would still have a ±15% uncertainty to cover the range found in the literature. Together, this exercise shows that not only can the relative error in the predictions be exceedingly large, but that it is largest precisely in the region of most interest for predicting the properties of the weld due to variation in residual stress and phase transformations. Furthermore, this variation is only due to the uncertainty of a single thermophysical parameter. This will undoubtedly grow when the variation in all parameters needed for full multiphysics models is considered.

In practice, modelers use these variations as guardrails—upper and lower bounds—on the parameters used for their models. These parameters are adjusted within these bounds based on comparisons of their model outputs to measured quantities through a validation process. Although this procedure is useful in practice, it does not allow one to rigorously test the capability of the model for predicting weld performance outside the narrow scope of experimental conditions for which the model is validated. The vast parameter space allowed by the variation in thermophysical properties allows a model to be tuned to give a satisfactory answer without ever knowing if the chosen parameters are accurate, which limits the model’s ability to be predictive. The larger context of the work presented here is that our measured material properties are directly linked to experimental data useful for laser weld model validation [1], which will allow for more rigorous testing of laser weld model predictions.

## Material and experimental methods

The material analyzed is an AISI 316 stainless steel. The exact composition is given in Table 1.

Two experimental systems were used to measure the thermophysical properties presented in this work. A subsecond ohmic pulse-heating apparatus (OPA) was used to measure temperature-dependent specific enthalpy, electrical resistivity, thermal radial expansion, and density. In addition, a commercial differential scanning calorimeter (DSC), the NETZSCH DSC 404 C Pegasus^1^, was used to obtain specific heat as a function of temperature. With the obtained DSC data, it is also possible to expand the low-end temperature range of the OPA data to room temperature.

### Ohmic pulse heating

The pulse-heating apparatus at Graz University of Technology was originally developed in the 1990s by Kaschnitz [9] and has been previously described in reference [10]. Wire-shaped samples with a diameter of 0.7 mm and a length of 60 mm are polished with abrasive paper (ISO Grit designation P1200), cleaned with acetone, and placed into a sample holder. This holder is then put into the electrical circuit containing a 500 μF capacitor bank and is placed inside a slightly pressurized (1.3 bar) nitrogen-filled chamber to prevent arcing. The chamber has optical access windows for a pyrometer and camera. The capacitor bank is charged to about 8 kV that is then applied to the wire. The experiment is initiated by a Krytron-triggered ignition. The same mechanism is used to precisely stop the experiment at a predefined time, by dissipating the residual voltage on the capacitor bank across a graphite resistor instead of over the (evaporated) sample. Depending on the ohmic resistance of the sample, the voltage drives a large current (up to 10 kA) through the wire, generating strong heating. Within 50 μs, the sample’s temperature rises past its melting point, through the liquid phase until it finally evaporates. One benefit of these short timescales is the inability of the wire to collapse due to the gravitational force in the liquid state. In fact, the liquid column stands vertically in the sample holder, expanding radially, until it explodes. A second benefit is it being quasi-containerless: The wire is clamped at two end points, and the short time scales suppress chemical reactions with these connections and its environment. Because very high currents are rapidly switched on and off, all measuring leads are shielded copper and lead into a Faraday room where the computer for data acquisition resides.

#### Temperature

Temperature measurements in our OPA system were performed with an optical pyrometer operating at a wavelength of 1569.5 nm. For accurate temperature measurements, it is necessary to know the material’s emissivity at the pyrometer measuring wavelength as a function of temperature. However, under the assumption that emissivity in the liquid phase stays constant, it is possible to calibrate the pyrometer by identifying the melting plateau in the pyrometer signal and assigning the known melting temperature to this value. Due to the sensitivity of the pyrometer photodiode (InGaAs), it is not possible to measure surface radiance below a sample temperature of 1100 K. Instead, we extend the range to lower temperatures by correlation with DSC results as will be explained later.

#### Enthalpy

Because of the short duration of the experiment, heat losses are negligible, and it is assumed that the electrical energy is completely converted into heat. Thus, it is possible to determine the supplied specific heat *Q*_S_(*t*) by integrating the electrical power according to

(3) $$Q_{\text{S}}(t)=\frac{1}{m}\cdot \int_{0}^{t}U\left(t^{\prime }\right)\cdot I\left(t^{\prime }\right)\;\text{d}t^{\prime },$$

where *m* is the mass of the specimen, *U*(*t*′) the voltage drop along the specimen at time *t*′, and *I*(*t*′) the current across the specimen at time *t*′. As pulse heating is an isobaric process, the specific enthalpy is given by Eq. (3). The voltage is measured by contacting the sample with two molybdenum knives, with a distance *l* between one other, and measuring two voltage drops to a common ground. The difference of these voltage drops yields the voltage drop *U*(*t*′) along the specimen. Current is measured inductively with a Pearson probe [11]. The mass is determined from the diameter *d* of the sample at room temperature measured with a laser micrometer, the distance between the voltage knives *l*, and the density at room temperature.

#### Electrical resistivity

The resistivity of a conducting material is defined as

(4) $$\rho (t)=R(t)\cdot \frac{A(t)}{l(t)},$$

with *R*(*t*) the time-dependent resistance, *A*(*t*) the time-dependent specimen cross-sectional area, and *l*(*t*) the time-dependent length of the sample. Note that because of the high heating rates, the length of the sample remains unaffected during the experiment, *l*(*t*) = *l*(*t*_0_) = *l*. According to Ohm’s law, Eq. (4) further yields

(5) $$\rho (t)=\frac{U(t)}{I(t)}\cdot \frac{d{\left(t\right)}^{2}\cdot \pi }{4\cdot l}.$$

It is useful to define the resistivity according to the samples initial geometry (IG) by

(6) $$\rho_{\text{IG}}(t)=\frac{U(t)}{I(t)}\cdot \frac{d_{\text{RT}}^{2}\cdot \pi }{4\cdot l_{\text{RT}}},$$

with *d*_RT_ and *l*_RT_ the diameter and distance between the voltage knives at room temperature (RT), respectively. Thus, resistivity including considerations of thermal expansion can be defined as

(7) $$\rho (t)=\rho_{\text{IG}}(t)\cdot {\left(\frac{d(t)}{d_{\text{RT}}}\right)}^{2}.$$

#### Thermal expansion and density

Thermal expansion was measured by obtaining shadow images of the expanding wire every 2.5 μs. To obtain the fast data processing rates for these experiments, a mechanically masked CCD chip was used. Only 8 pixel-rows (with 384 pixels each) of the chip were exposed, leaving the remainder of the chip as a fast buffer storage. Therefore, it was possible to obtain up to 10 images of a small cross section of the expanding wire during an experiment. The unheated wire, with a diameter of 0.7 mm, occupies approximately 140 pixels per row. The spatial resolution is approximately 0.6 pixels. Before the experiment was started, a set of pictures of the cold wire was taken. Summing over the lines of each obtained picture produces an intensity profile from which the diameter of the wire was determined by taking the full width at half maximum (FWHM) of the intensity profile. All measured quantities shared the same time basis due to a common trigger pulse, and a temperature was assignable to each of the obtained pictures. The volume expansion as a function of temperature was then calculated by the ratio of the FWHM value of the hot wire at a certain time and temperature, *d*(*T*), to the FWHM value of the cold wire *d*_0_

(8) $$\frac{V(T)}{V_{0}}={\left(\frac{d(T)}{d_{0}}\right)}^{2}.$$

Equation (8) is only true when longitudinal expansion of the wire is prevented and only radial expansion of the wire occurs. In pulse-heating experiments, this is the case as shown by Huepf [12].

Density as a function of temperature *D*(*T*) can then be derived by combining the density at room temperature *D*_0_ with the volume expansion

(9) $$D(T)=D_{0}\cdot {\left(\frac{d_{0}}{d(T)}\right)}^{2}$$

with a more detailed explanation found in [13].

### Differential scanning calorimeter (DSC)

To measure specific heat capacity in the solid phase and extend the temperature range of the OPA data, a commercial DSC, the NETZSCH DSC 404 C Pegasus, was used. The DSC measures the temperature difference between two crucibles. For one DSC experiment, a total of three measurements were performed: One with two empty crucibles to determine the baseline, a run with one empty crucible and a reference material in the other, and finally a run with one empty crucible and the sample material. As a result of the specific heat capacity of the material (reference or sample), there is a temperature gradient between the empty crucible and the filled one, when heating up both equally. By measuring the temperature gradient for a reference material with a known specific heat capacity, it is possible to determine the specific heat capacity of the sample under test. Ideally, the temperature difference between two empty crucibles would be zero. However, due to minor imperfections in the alignment of the measuring system and unequal masses of the crucibles this is not exactly true. Thus, it is necessary to measure the baseline and subtract it from both the reference measurements, as well as the sample measurement. The specific heat capacity *c*_p,S_ of the sample was determined by the equation

(10) $$c_{\text{p},\text{S}}(T)=c_{\text{p},\text{R}}(T)\cdot \frac{m_{\text{R}}}{m_{\text{S}}}\frac{\phi_{\text{S}}-\phi_{\text{B}}}{\phi_{\text{R}}-\phi_{\text{B}}},$$

with *T* the temperature, *c*_p,R_(*T*) the specific heat capacity of the reference material, *m*_R_ the mass of the reference, *m*_S_ the mass of the sample, and *ϕ*_*i*_ the DSC signals (*i* = R,S,B; R stands for reference, S for sample, and B for baseline).

The temperature range of the DSC measurements starts at 473 K. Enthalpy can be calculated from *c*_p,S_ by integrating heat capacity with respect to temperature:

(11) $$H(T)=\int_{473\;\text{K}}^{T}c_{\text{p},\text{S}}\left(T^{\prime }\right)\text{d}T^{\prime }+(473\;\text{K}-298\;\text{K})\cdot c_{\text{p},\text{S}}(473\;\text{K}).$$

**Figure 3:**
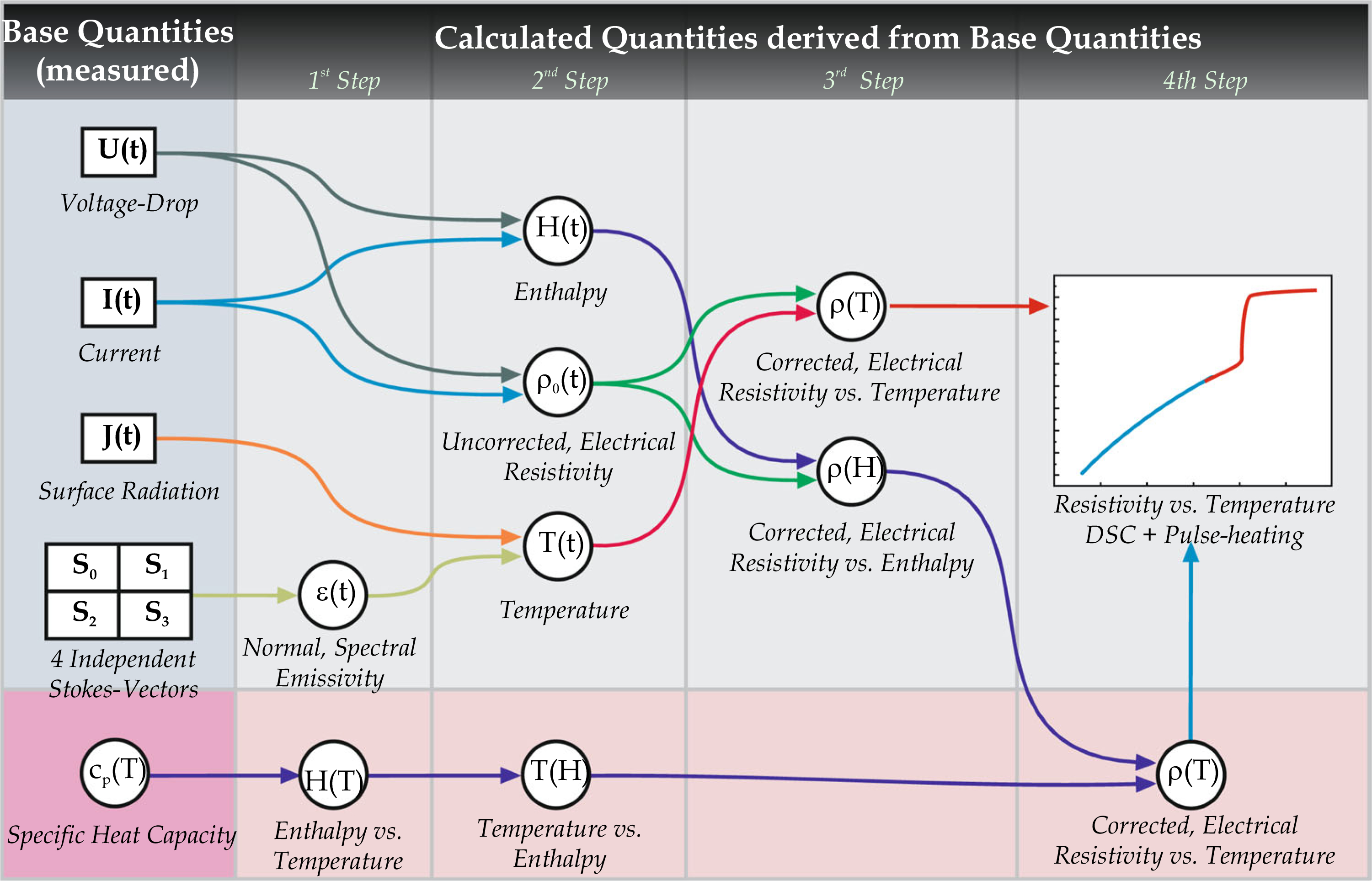
Flowchart on how to expand the temperature range for the OPA measurements with the help of a DSC.

By obtaining enthalpy as a function of temperature, *H*(*T*), it is then possible to determine the inverse: Temperature as a function of enthalpy *T*(*H*). As enthalpy data obtained by OPA measurements start from room temperature, these data can be matched with the enthalpy data obtained from DSC measurements to assign a temperature to the enthalpy data from OPA measurements. This is presented graphically in Fig. 3.

To determine the density at room temperature, cylinders with a diameter of *d* = (10.98 ± 001) × 10^−3^m and a height of *h* = (5.19 ± 0.01) × 10^−3^m were machined. The mass of the cylinder was measured with a Mettler Toledo PB303 balance as *m* = (3.883 ± 0.001) × 10^−3^kg, yielding a room temperature density of (7904 ± 25) kg m^−3^.

## Results

**Figure 4:**
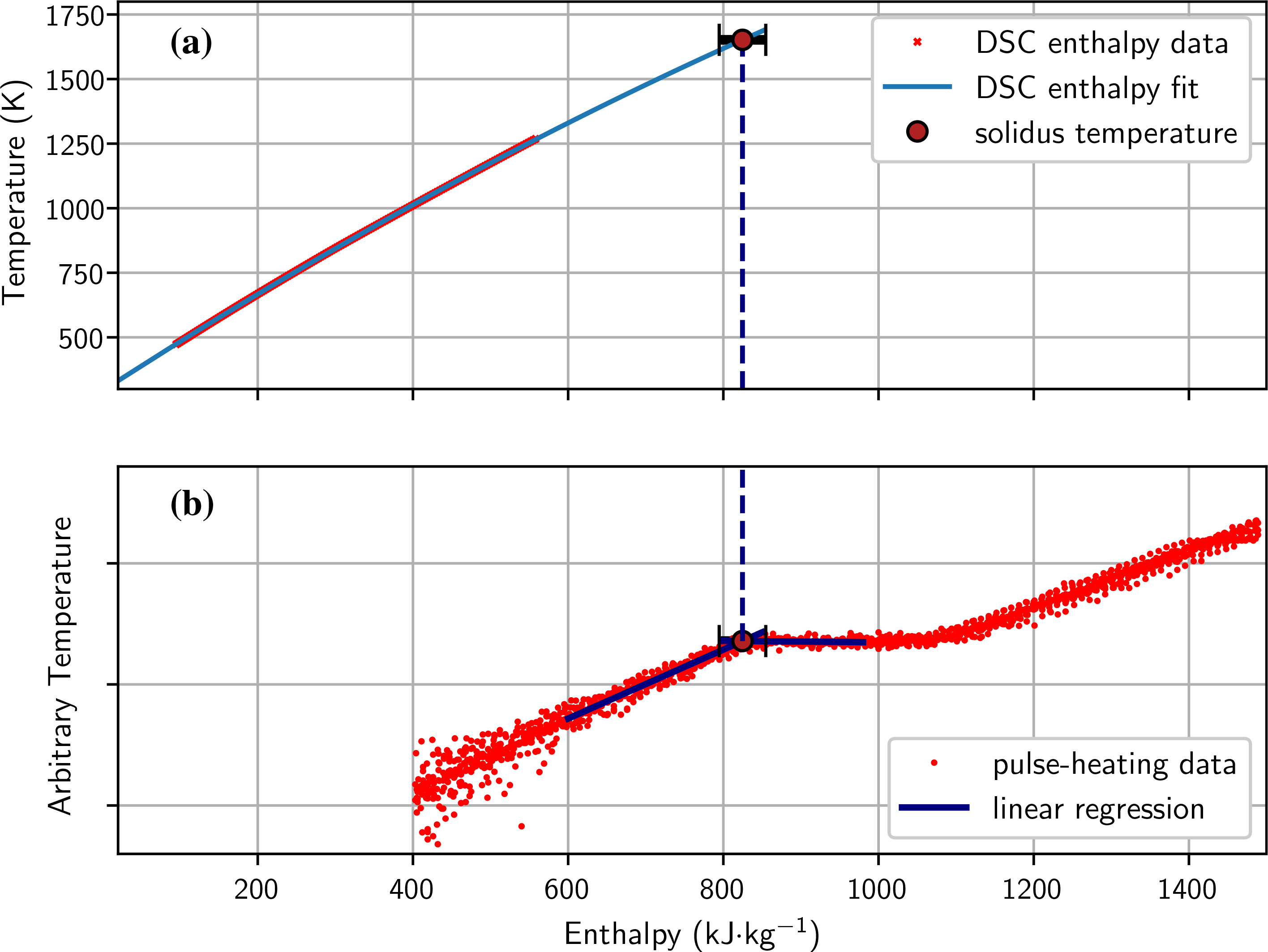
Method to obtain solidus temperature by matching enthalpy of pulse-heating data to enthalpy of DSC measurements. **a** Shows the temperature as a function of enthalpy from DSC measurements, which has been fit and extrapolated. **b** Shows the pyrometer signal as a function of enthalpy from pulse-heating measurements. The onset of the melting plateau has been determined by linearly fitting the plateau as well as the rising pyrometer signal before the plateau. The intersection of those fits yields the onset of the melting plateau. The corresponding temperature to this enthalpy value was then determined by extrapolating DSC enthalpy data.

Solidus (*T*_s_) and liquidus (*T*_l_) temperatures of the material were determined by DSC measurements and are presented in Table 2. Due to evaporation of the material in the melt phase during DSC measurements, the onset of the melting peak in the DSC signal provides an upper limit of the solidus temperature. By repeated heating of a sample in the DSC, it was found that the onset of the melting peak was shifted to higher temperatures compared to the first heating. Manganese, which has a very high vapor pressure, evaporates from the material during the initial heating, altering the solidus temperature by approx. 20 K. Therefore, the solidus temperature was determined by taking the mean of the onset of the melting peak from the DSC measurements and a temperature found by matching the enthalpy of the OPA data to the extrapolated enthalpy of the DSC measurements (see Fig. 4).

The higher uncertainty of the liquidus temperature is due to the strong evaporation of manganese and chromium at melting. Therefore, as the composition of the material changes during melting, the liquidus temperature determined by identifying the endothermal peak in the DSC heating curve is not the true liquidus temperature of the initial material. Note: Samples used in the DSC were not reused for subsequent OPA measurements.

**Figure 5:**
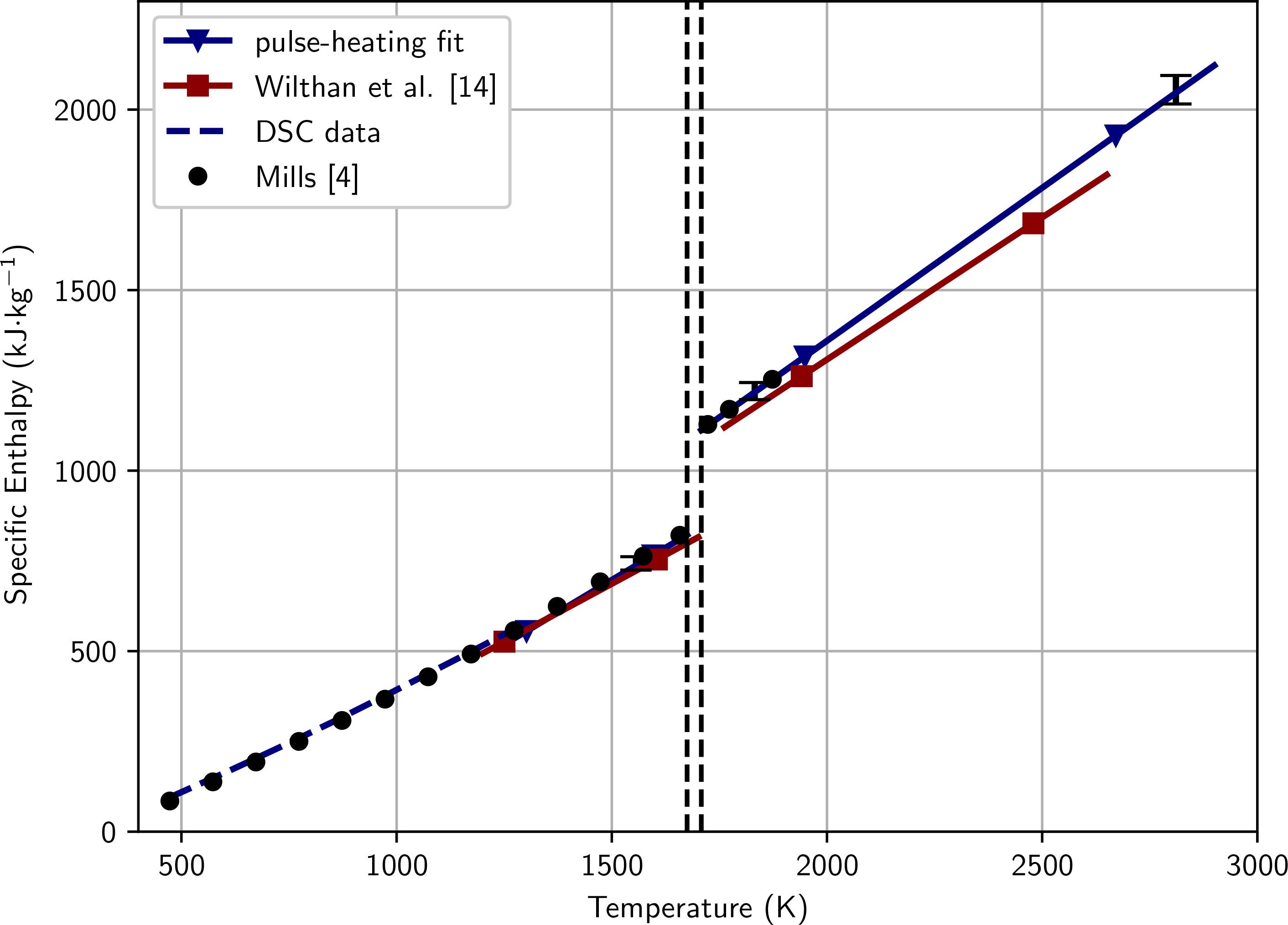
Specific enthalpy as a function of temperature. The reference temperature for enthalpy measurements is room temperature (298 K). The results of this work are compared to measurements by Wilthan et al. [14], who measured a similar AISI 316L stainless steel as well as to the recommended values by Mills [4], who also presents values for a different, but similar AISI 316L stainless steel. The horizontal dashed lines give solidus and liquidus temperatures.

The specific enthalpy *H*_298_(*T*) = *H*_s_(*T*) – *H*(298 K) as a function of temperature *T* is shown in Fig. 5. The fitting coefficients are given in Table 3. The horizontal dashed lines in Fig. 5 represent the beginning and end of the melting. The difference in enthalpy between the ending of the solid phase (*H*_298,1_ = 822 kJ kg^−1^) and beginning of the liquid phase (*H*_298,2_ = 1112 kJ kg^−1^) gives the latent heat of fusion *ΔH* = 290 kJ kg^−1^.

**Figure 6:**
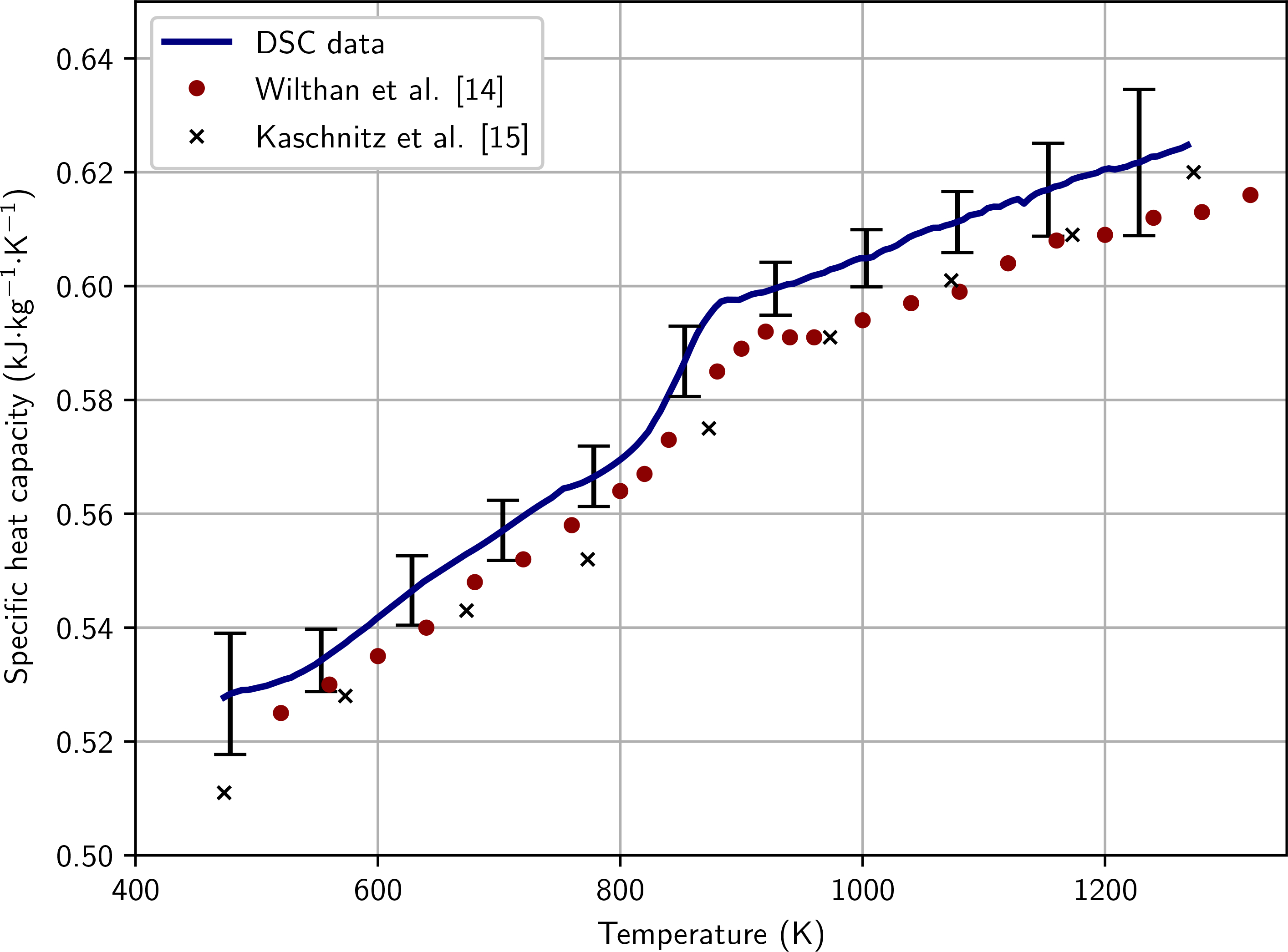
Specific heat capacity obtained via DSC measurements. The solid blue curve represents this work. The red circles represent measurements of a AISI 316L stainless steel, by Wilthan et al. [14]. The black crosses represent measurements of a similar AISI 316L stainless steel by Kaschnitz et al. [15].

In the liquid phase and at the end of the solid phase, specific heat capacity *c*_p_ is represented by the slope of *H*_298_(*T*), yielding *c*_p_ = 0.847 kJ kg^−1^ K^−1^ for the liquid phase (1708 K to 2900 K) and *c*_p_ = 0.714 kJ kg^−1^ K^−1^ for the end of the solid phase. Furthermore, the specific heat capacity in the solid phase has been measured with DSC with the results given in Fig. 6 and in Table 7. DSC-measured specific heat capacity ranges from 500 K to 1250 K. There is a slight kink in the data at 820 K, which most likely results from precipitates dissolving in the material.

**Figure 7:**
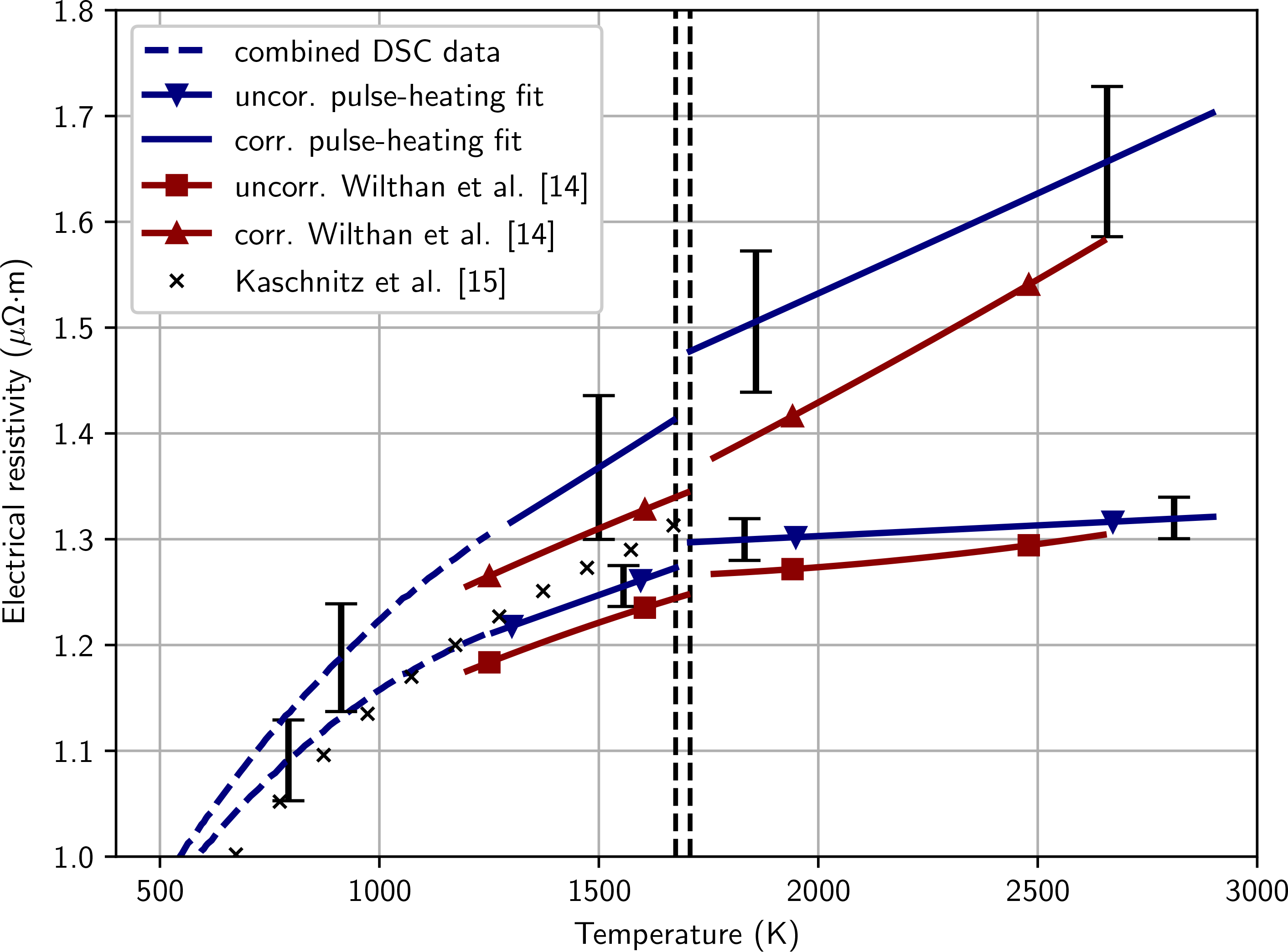
Electrical resistivity as a function of temperature. The solid blue line with triangle-shaped markers represents this work, assuming the initial geometry of the sample. The solid blue line represents this work’s resistivity values corrected for thermal expansion. The red lines represent measurements of a AISI 316L stainless steel by Wilthan et al. [14]. The black crosses represent measurements of a similar AISI 316L stainless steel by Kaschnitz et al. [15].

Figure 7 shows the results for electrical resistivity as a function of temperature both with and without correction for the change of volume due to thermal expansion. The solid blue line with triangle-shaped markers represents the linear fits of the pulse-heating data using the initial sample geometry. The solid blue lines without markers are the linear fit of the pulse-heating data corrected for volume expansion. The dashed blue lines represent the fitted pulse-heating data with matched temperatures from DSC measurements. The uncorrected resistivity is represented by the lower line; the corrected resistivity is represented by the upper line. The coefficients for the polynomial fits are given in Table 3.

**Figure 8:**
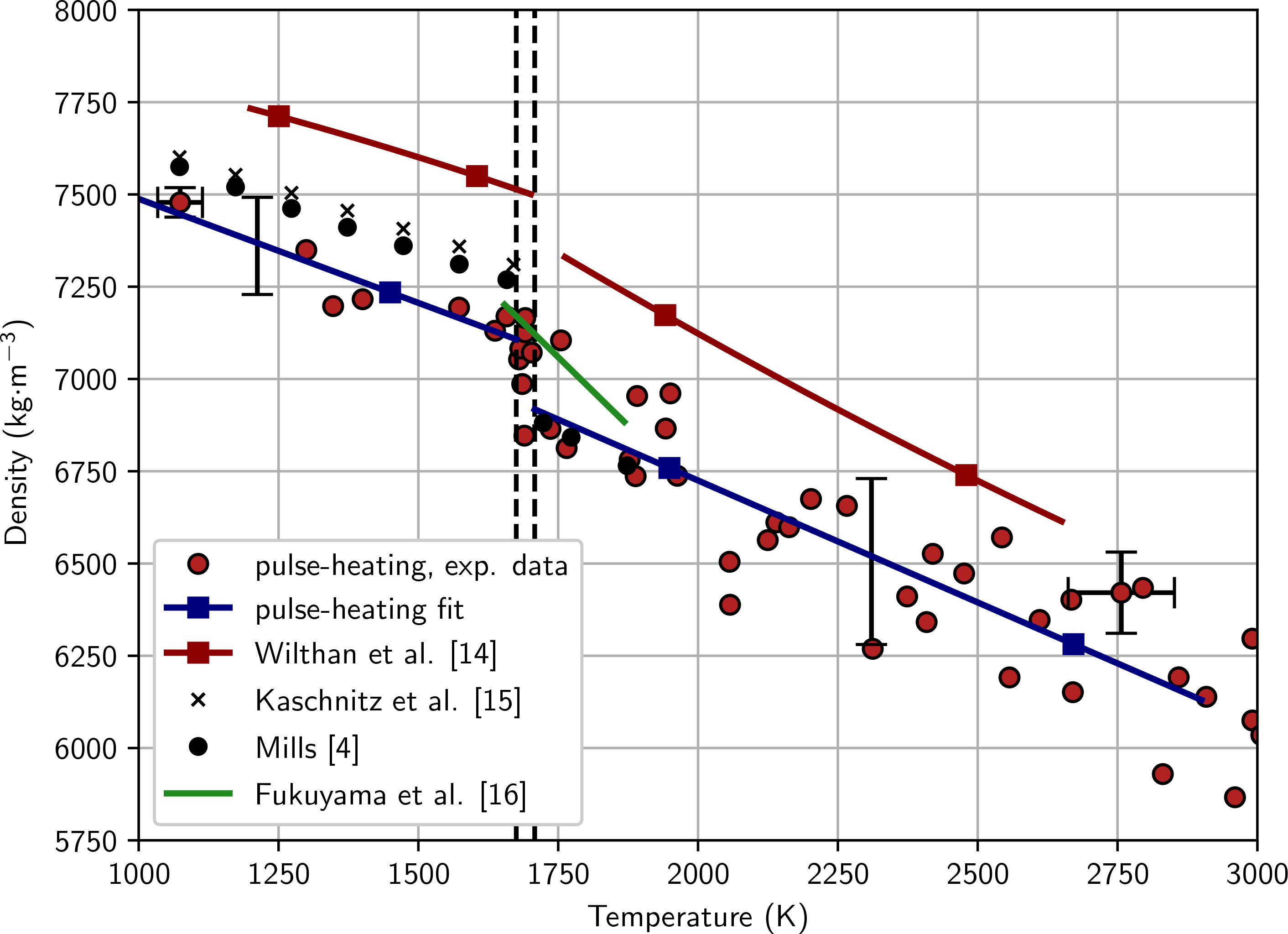
Density as a function of temperature. The red circles are the measured data, and the solid blue lines are linear fits. Black circles represent the recommended values of a similar steel by Mills [4]. The solid red lines are data for a AISI 316L stainless steel measured by Wilthan et al. [14]. The black crosses represent data of a similar AISI 316L stainless steel measured by Kaschnitz et al. [15]. The solid green line without markers represents data of a similar AISI 316L stainless steel measured by Fukuyama et al. [16].

Density as a function of temperature is presented in Fig. 8. The red circles are actual measurement points with the solid blue lines the linear fits. The coefficients and their respective temperature ranges are given in Table 3.

### Uncertainties

The uncertainties of the DSC measurements were assessed statistically. Other uncertainties were calculated according to the Guide to the Expression of Uncertainty in Measurement (GUM) [17]. Uncertainties of the fitting coefficients were calculated following the guide by Matus [18]. Uncertainty values are listed in Table 4.

Uncertainty values for the temperature-dependent density were assessed by evaluating the radii of the hot and cold wire 10 times to obtain a statistical uncertainty. As this only accounts for uncertainty in evaluation, the standard deviation of the obtained radii was then doubled.

## Conclusion

Thermophysical properties of NIST SRM for 316L stainless steel (1155a), a Cr18–Ni12–Mo2 steel, were measured by means of ohmic pulse heating in combination with a differential scanning calorimeter (DSC). These properties include specific heat capacity, specific enthalpy, corrected and uncorrected electrical resistivity, as well as density, all as a function of temperature. Our results were compared to the literature values of similar steels, as there are no data available in the literature for this exact SRM. Enthalpy and uncorrected resistivity are in good agreement to the literature. Specific heat capacity in the liquid phase obtained in our work is 7% higher than reported by Wilthan et al. [14] and 8.5% higher than reported by Fukuyama et al. [16]. The value for specific heat capacity in the liquid phase reported by Mills [4] is 2% lower than the obtained value of this work. Density in the liquid phase is in good agreement to the values reported by Mills [4]. Our density data are 5% lower, compared to the data reported by Wilthan et al. [14]. The density is indirectly proportional to the thermal volume expansion of the material (see (9)). Therefore, electrical resistivity, corrected for thermal expansion, is 5% higher, compared to the values reported by Wilthan et al. [14]. However, it has to be noted that the composition of the AISI 316L steels reported in the literature differs from the composition of the NIST SRM 1155a. The exact compositions of the samples are shown in Table 5. The literature data are reported as comparison values only. Uncertainty assessment was performed according to the GUM. Thermophysical property data obtained in this work are listed in Tables 6 and 7. Coefficients for all fits are given in Table 3. Results of the uncertainty assessment are listed in Table 4.

## Figures and Tables

**Table 1:** Certified mass fraction values for SRM 1155a [8]

Element	Mass fraction/%	Coverage factor, *k*

Carbon (C)	0.0260 ± 0.0036	2.45
Cobalt (Co)	0.225 ± 0.018	2.26
Chromium (Cr)	17.803 ± 0.099	2.20
Copper (Cu)	0.2431 ± 0.0050	2.20
Iron (Fe)	64.71 ± 0.12	2.00
Manganese (Mn)	1.593 ± 0.060	2.06
Molybdenum (Mo)	2.188 ± 0.015	2.18
Niobium (Nb)	0.0082 ± 0.0014	3.18
Nickel (Ni)	12.471 ± 0.056	2.20
Phosphorus (P)	0.0271 ± 0.0012	2.11
Silicon (Si)	0.521 ± 0.017	2.03
Titanium (Ti)	0.0039 ± 0.0012	2.45
Vanadium (V)	0.0725 ± 0.0046	2.23
Tungsten (W)	0.0809 ± 0.0059	2.45
Oxygen (O)^a^	0.003	

a Oxygen content was estimated from one or more NIST or collaborator test methods

**Table 2:** Solidus and liquidus temperatura of SRM 1155a

	*T*/K	*ΔT*/K

*T*_s_	1675	15
*T*_l_	1708	30

*T* is the temperatura, and *ΔT* is the temperatura uncertainty (*k* = 2)

**Table 3:** Polynomial fit coefficients for the thermophysical properties according to *y* = *a* + *bT* + *cT*^2^

Property *y*	Unit	*a*	*b*	*c*	Range *T*/K	State	Method

*D*(*T*)	kgm^−3^	8052	− 0.564		500 ≤ *T* ≤ *T*_s_	s	OPA
*D*(*T*)	kgm^−3^	8065	− 0.661		*T*_l_ ≤ *T* ≤ 2900	l	OPA
*ρ*_IG_(*T*)	μΩ m	0.624	7.951 × 10^−4^	−2.61 × 10^−7^	500 ≤ *T* ≤ 1250	s	DSC + OPA
*ρ*_IG_(*T*)	μΩ m	1.026	1.477 × 10^−4^		1350 ≤ *T* ≤ *T*_s_	s	OPA
*ρ*_IG_(*T*)	μΩ m	1.263	2.021 × 10^−5^		*T*_l_ ≤ *T* ≤ 2900	l	OPA
*ρ*_corr_(*T*)	μΩ m	0.977	2.605 × 10^−4^		1350 ≤ *T* ≤ *T*_s_	s	OPA
*ρ*_corr_(*T*)	μΩ m	1.154	1.893 × 10^−4^		*T*_l_ ≤ *T* ≤ 2900	l	OPA
*H*_s_(*T*)	kJkg^−1^	− 139	0.459	7.16 × 10^−5^	500 ≤ *T* ≤ 1250	s	DSC
*H*_s_(*T*)	kJkg^−1^	− 374	0.714		1350 ≤ *T* ≤ *T*_s_	s	OPA
*H*_s_(*T*)	kJkg^−1^	− 335	0.847		*T*_l_ ≤ *T* ≤ 2900	l	OPA

*T*_s_ = 1675 K, *T*_1_ = 1708K, are the solidus and liquidus temperatures, respectively. *D* is the density, *ρ*_IG_ is the electrical resistivity assuming initial geometry, *ρ*_corr_ is the electrical resistivity corrected for thermal expansion, *H*_s_ is the specific enthalpy, and *T* is the temperature. *S* and *l* denote data in solid and liquid phase

**Table 4:** Assessed uncertainties of the measured thermophysical properties

Property *y*	Unit	Uncertainty (%)	State	Method

*D*(*T*)	kgm^−3^	2.5	Solid	OPA
*D*(*T*)	kgm^−3^	3	Liquid	OPA
*ρ*_IG_(*T*)	μΩ m	3	Solid	DSC + OPA
*ρ*_IG_(*T*)	μΩ m	2.5	Solid	OPA
*ρ*_IG_(*T*)	μΩ m	2.5	Liquid	OPA
*ρ*_corr_(*T*)	μΩ m	5	Solid	OPA + DSC
*ρ*_corr_(*T*)	μΩ m	5.5	Solid	OPA
*ρ*_corr_(*T*)	μΩ m	4.5	Liquid	OPA
*H*_s_(*T*)	kJkg^−1^	2.5	Solid	OPA
*H*_s_(*T*)	kJkg^−1^	2.9	Liquid	OPA
*c*_p_(*T*)*	kJkg^−1^K^−1^	1.8–3	Solid	DSC

If not indicated with an asterisk, all uncertainty values are calculated according to the GUM with a coverage factor *k* = 2. Asterisk: uncertainty value assessed statistically

**Table 5:** Composition of the AISI 316L stainless steels compared in mass %

References	Fe	Cr	Ni	Mo	Mn	Si	C

SRM	bal.	17.803	12.471	2.188	1.593	0.521	0.026
Wilthan et al. [14]	bal.	17.50	14.50	2.70	1.70	0.30	0.030
Kaschnitz et al. [15]	bal.	16.7	10.1	2.0	1.63	0.41	0.03
Mills [4]	bal.	17	12	2.5	2	1	0.08
Fukuyama et al. [16]	bal.	17.3	12.0	2.23	1.61	0.53	0.015

**Table 6:** Collected thermophysical data of this work

*T*/K	*H*_S_/kJ kg^−1^	*ρ*_IG_/μΩ m	*ρ*_corr_/μΩ m	*D*(*T*)/kg m^−3^	$\frac{V(T)}{V_{0}}/1$

500	109	0.954	0.970	7770	1.017
600	163	1.007	1.033	7714	1.025
700	218	1.054	1.089	7657	1.033
800	274	1.093	1.138	7601	1.041
900	333	1.128	1.183	7544	1.049
1000	393	1.157	1.223	7488	1.057
1100	454	1.182	1.259	7432	1.065
1200	515	1.203	1.290	7375	1.073
1300	554	1.218	1.316	7319	1.081
1400	626	1.232	1.342	7263	1.089
1500	697	1.247	1.368	7206	1.097
1600	769	1.262	1.394	7150	1.105
1700	1042	1.291	1.464	6964	1.132
1800	1190	1.299	1.495	6857	1.151
1900	1275	1.301	1.514	6791	1.163
2000	1360	1.303	1.532	6725	1.176
2100	1444	1.305	1.551	6659	1.189
2200	1529	1.307	1.570	6593	1.201
2300	1614	1.309	1.589	6527	1.214
2400	1699	1.311	1.608	6461	1.226
2500	1783	1.313	1.627	6395	1.239
2600	1868	1.315	1.646	6329	1.251
2700	1953	1.317	1.665	6262	1.264
2800	2037	1.319	1.684	6196	1.277

*D* is the density, *ρ*_IG_ is the electrical resistivity, assuming initial geometry, *ρ*_corr_ is the electrical resistivity, corrected for thermal expansion, *H*_s_ is the specific enthalpy, $\frac{V(T)}{V_{0}}$ is the thermal expansion, and *T* is the temperature

**Table 7:** Specific heat capacity *c*_p_ as a function of temperature *T* determined via DSC

*T* K	*c*_p_ kJ kg^−1^ K^−1^	*Δ*(*c*_p_) kJ kg^−1^ K^−1^	*T* K	*c*_p_ kJ kg^−1^ K^−1^	*Δ*(*c*_p_) kJ kg^−1^ K^−1^

473	0.528	0.011	873	0.595	0.005
493	0.529	0.009	893	0.598	0.005
513	0.530	0.007	913	0.599	0.005
533	0.532	0.006	933	0.600	0.005
553	0.534	0.005	953	0.601	0.004
573	0.537	0.006	973	0.603	0.004
593	0.541	0.006	993	0.605	0.005
613	0.544	0.006	1013	0.606	0.005
633	0.547	0.006	1033	0.608	0.005
653	0.550	0.006	1053	0.610	0.005
673	0.553	0.006	1073	0.611	0.005
693	0.556	0.005	1093	0.613	0.006
713	0.559	0.005	1113	0.614	0.006
733	0.561	0.005	1133	0.615	0.006
753	0.564	0.005	1153	0.617	0.008
773	0.566	0.005	1173	0.619	0.009
793	0.569	0.006	1193	0.620	0.010
813	0.572	0.006	1213	0.621	0.012
833	0.578	0.007	1233	0.622	0.013
853	0.587	0.006	1253	0.624	0.015

*Δc*_p_ denotes the *k* = 2 uncertainty

## Footnotes

1 Certain commercial equipment, software, and/or materials are identified in this paper in order to adequately specify the experimental procedure. In no case does such identification imply recommendation or endorsement by the National Institute of Standards and Technology, nor does it imply that the equipment and/or materials used are necessarily the best available for the purpose.

## References

[1] Simonds BJ, Sowards J, Hadler J, Pfeif E, Wilthan B, Tanner J, Harris C, Williams P (2018) Time-resolved absorptance and melt pool dynamics during intense laser irradiation of a metal. Phys Rev Appl 10(4):044061. 10.1103/PhysRevApplied.10.044061 PMC7047776 32118095

[2] Jenkins R, Westover R (1962) Thermal diffusivity of stainless steel from 20° to 1000° C. J Chem Eng Data 7(3):434–437. 10.1021/je60014a038

[3] Lucks C, Deem H (1958) Thermal properties of 13 metals: thermal properties of 13 metals. ASTM 9:795–795. 10.1002/maco.19580091216

[4] Mills K (2002) Recommended values of thermophysical properties for selected commercial alloys. Woodhead Publishing, Sswston

[5] Specification for Chromium and Chromium-Nickel Stainless Steel Plate, Sheet, and Strip for Pressure Vessels and for General Applications. 10.1520/a0240_a0240m-17

[6] Mills K, Su Y, Li Z, Brooks R (2004) Equations for the calculation of the thermo-physical properties of stainless steel. ISIJ Int 44(10):1661–1668. 10.2355/isijinternational.44.1661

[7] Lippold L (1994) Solidification behavior and cracking susceptibility of pulsed-laser welds in austenitic stainless steels. Weld J 73(6):129–139

[8] SRM 1155a (2013) AISI 316 Stainless Steel. National Institute of Standards and Technology; U.S. Department of Commerce, Gaithersburg, MD

[9] Kaschnitz E, Pottlacher G, Jaeger H (1992) A new microsecond pulse-heating system to investigate thermophysical properties of solid and liquid metals. Int J Thermophys 13(4):699–710. 10.1007/bf00501950

[10] Leitner M, Leitner T, Schmon A, Aziz K, Pottlacher G (2017) Thermophysical properties of liquid aluminum. Metall Mater Trans A 48:3036–3045. 10.1007/s11661-017-4053-6

[11] Pearson Electronics I (2019) Pearson current monitor model 3025. http://www.pearsonelectronics.com/pdf/3025.pdf. Accessed 05 Apr

[12] Huepf T (2010) Density determination of liquid metals: Ph.D. thesis, Graz University of Technology

[13] Leitner M, Schroer W, Pottlacher G (2018) Density of liquid tantalum and estimation of critical point data. Int J Thermophys 39(11):124. 10.1007/s10765-018-2439-3

[14] Wilthan B, Reschab H, Tanzer R, Schuetzenhoefer W, Pottlacher G (2008) Thermophysical properties of a chromium–nickel–molybdenum steel in the solid and liquid phases. Int J Thermophys 29(1):434–444. 10.1007/s10765-007-0300-1

[15] Kaschnitz E, Kaschnitz H, Schleutker T, Guelhan A Bonvoisin B (2017) Electrical resistivity measured by millisecond pulse-heating in comparison to thermal conductivity of the stainless steel AISI 316L at elevated temperature. High Temp High Press 46

[16] Fukuyama H, Higashi H, Yamano H (2019) Thermophysical properties of molten stainless steel containing 5 mass% B4C. Nucl Technol 0(0):1–10. 10.1080/00295450.2019.1578572

[17] Joint Committee for Guides in Metrology (JCGM/WG 1), WG (ed) (1993) Guide to the expression of uncertainty in measurement: BIPM

[18] Matus M (2005) Koeffizienten und Ausgleichsrechnung: Die Messunsicherheit nach GUM. Teil 1: Ausgleichsgeraden (Coefficients and adjustment calculations: measurement uncertainty under GUM. Part 1: best fit straight lines): tm—Technisches Messen 72(10/2005). 10.1524/teme.2005.72.10_2005.584

